# Analysis of Oral Microbiota in Herpetiform Aphthous Ulcers Patients

**DOI:** 10.3290/j.ohpd.c_2196

**Published:** 2025-08-19

**Authors:** Guoqing Wang, Xin Tong, Chenhong Zhang, Ran Zhuo, Chenlu Liu, Cuihuan Wang, Mengge Hao, Li Ren

**Affiliations:** a Guoqing Wang Professor, Department of Clinical Laboratory, Tianjin Stomatological Hospital, School of Medicine, NanKai University, Tianjin, China; Tianjin Key Laboratory of Oral and Maxillofacial Function Reconstruction, Tianjin, China. Study design. *Equal first author with Tong.; b Xin Tong Lecturer, Department of Clinical Laboratory, Tianjin Stomatological Hospital, School of Medicine, NanKai University, Tianjin, China; Tianjin Key Laboratory of Oral and Maxillofacial Function Reconstruction, Tianjin, China; Department of Clinical Laboratory, Tianjin Medical University Cancer Institute and Hospital, Tianjin, China; National Clinical Research Center for Cancer, Tianjin, China; Key Laboratory of Cancer Prevention and Therapy, Tianjin, China; Tianjin’s Clinical Research Center for Cancer, Tianjin, China. Wrote the manuscript. *Equal first author with Guoqing Wang; c Chenhong Zhang Laboratory technician, Department of Clinical Laboratory, Tianjin Stomatological Hospital, School of Medicine, NanKai University, Tianjin, China; Tianjin Key Laboratory of Oral and Maxillofacial Function Reconstruction, Tianjin, China. Collected samples. **Equal second author with Zhuo.; d Ran Zhuo Laboratory technician, Department of Clinical Laboratory, Tianjin Stomatological Hospital, School of Medicine, NanKai University, Tianjin, China; Tianjin Key Laboratory of Oral and Maxillofacial Function Reconstruction, Tianjin, China. Collected samples. **Equal second author with Zhang.; e Chenlu Liu Professor, Department of Clinical Laboratory, Tianjin Stomatological Hospital, School of Medicine, NanKai University, Tianjin, China; Tianjin Key Laboratory of Oral and Maxillofacial Function Reconstruction, Tianjin, China. ***Equal third author. Provided clinical samples and patient information.; f Cuihuan Wang Lecturer, Department of Clinical Laboratory, Tianjin Stomatological Hospital, School of Medicine, NanKai University, Tianjin, China; Tianjin Key Laboratory of Oral and Maxillofacial Function Reconstruction, Tianjin, China. ***Equal third author. Performed statistical evaluations.; g Mengge Hao Laboratory Technician, Department of Clinical Laboratory, Tianjin Stomatological Hospital, School of Medicine, NanKai University, Tianjin, China; Tianjin Key Laboratory of Oral and Maxillofacial Function Reconstruction, Tianjin, China. ***Equal third author. Collected and analyzed data.; h Li Ren Professor, Department of Clinical Laboratory, Tianjin Medical University Cancer Institute and Hospital, Tianjin, China; National Clinical Research Center for Cancer, Tianjin, China; Key Laboratory of Cancer Prevention and Therapy, Tianjin, China; Tianjin’s Clinical Research Center for Cancer, Tianjin, China. Revised the manuscript.

**Keywords:** 16R rRNA, herpetiform aphthous ulcers, oral microbiota, recurrent aphthous ulcers.

## Abstract

**Purpose:**

To examine the microbiota in the oral mucosa and saliva of patients with herpetiform aphthous ulcers (HAU) and compare it with healthy individuals.

**Materials and Methods:**

16S rRNA sequencing was employed to analyze the oral mucosal bacterial communities of healthy individuals (healthy controls) and HAU patients (ulcerated sites, healthy sites, and healed ulcer sites).

**Results:**

Species richness in patients with HAU was statistically significantly lower than in healthy individuals. At the phylum level, the abundance of Firmicutes in the healthy sites of HAU patients was lower, while that of Proteobacteria was higher compared to healthy controls. In the ulcerated sites, the abundance of Firmicutes diminished, and the abundance of Proteobacteria increased relative to the healthy sites. In the healed ulcer sites, the abundance of these two phyla had partially recovered but had not yet reached the level of healthy sites in the ulcer phase. At the genus level, the abundance of Streptococcus in the healthy sites of HAU patients was lower than that in healthy controls, whereas Haemophilus_D was higher. In the ulcerated sites, the abundance of Streptococcus decreased, while the abundances of Neisseria and Haemophilus_D increased compared to the healthy sites. In the healed ulcer sites, the abundance of these three bacterial genera recovered to levels close to those in healthy sites during the ulcer phase. LEfSe analysis indicated that o_Enterobacterales_A, f_Pasteurellaceae, f_Erysipelotrichaceae, g_Bulleidia, f_Peptoniphilaceae, and g_Parvimonas were identified as biomarkers in the ulcerated sites.

**Conclusion:**

These findings highlight the distinct microbial signatures associated with HAU and suggest that microbial community changes may play a role in disease progression and healing.

Recurrent aphthous ulcers (RAU) are the most prevalent oral mucosal disease, with an incidence of 20% in the general population.^6^ It is characterized by multiple recurrent inflammatory ulcers that are circular or oval in shape with yellow or gray borders and erythematous edges and base.^11^ The main clinical manifestations of RAU include minor RAU, major RAU, and herpetiform aphthous ulcers (HAU). RAU can cause severe pain and severe difficulties in eating, drinking, swallowing, and speaking, thereby negatively affecting the quality of life of RAU patients.^1^ Current treatment options for RAU primarily involve the topical application of hormones and immunosuppressants, which only mitigate the severity of the ulcers without preventing their recurrence.^2,17^ Consequently, a comprehensive understanding of the etiology and pathogenesis of RAU is crucial for the development of more effective treatment strategies.

Changes in the oral mucosal microbiome have been noted as contributing factors to the development of persistent mucosal inflammatory conditions.^15^ Earlier research indicated that disruptions in the oral microbiota might be involved in the etiopathogenesis of RAU.^4^ Moreover, patients suffering from RAU exhibit differences in their oral microbiota compared to healthy individuals.^9^ The bacterium *Helicobacter pylori* could play a role in the development of RAU.^18^ It was found that the counts of *Veillonella* and *Streptococcus* were statistically significantly lower during the ulcerative stages of the RAU group compared to the non-ulcerative stages; furthermore, the occurrence of RAU was inversely associated with the *Veillonella dispar *count.^25^ Additionally, several infectious agents, including the genera *Actinobacillus, Haemophilus, Prevotella*, and *Vibrio*, were found to be elevated in cases of RAU.^27^ A recent investigation indicated that the occurrence of RAU is notably linked to an increase in *Escherichia coli* and a reduction in *Alloprevotella* abundances.^24^ However, the specific bacterial species associated with RAU have not been identified.

Metagenomic sequencing offers enhanced genome coverage and enables the acquisition of information regarding genetic diversity, molecular ecology, and microbial functions.^7^ Nonetheless, there have been limited metagenomic investigations focusing on HAU. Therefore, this study aimed to characterize the oral mucosal microbiota at ulcerated sites, healthy sites, and healed ulcer sites in patients with HAU.

## MATERIALS AND METHODS

### Ethics Statement

All experiments were approved by the Ethics Committee of the Tianjin Stomatological Hospital, conforming to the declaration of Helsinki, and informed consent was obtained from all subjects.

### Patient Recruitment Method

Six patients with HAU who were admitted to the Mucosal Department of Tianjin Stomatological Hospital were randomly selected for this study. The inclusion criteria consisted of patients experiencing oral ulcers at least once a month, without any other oral mucosal (including those caused by trauma) or systemic diseases. Exclusion criteria included: patients with abnormal routine blood tests (liver function, renal function, folic acid, vitamin B12, ferritin, zinc, T lymphocyte subsets, B cells, NK cells, IgA, IgG, IgM, IgE, C3, C4, and C-reactive protein); patients with a *Candida* culture count exceeding 10^3^ CFU/ml; smokers; individuals who had used antibiotics within the past three months; alcohol consumers; those with other oral mucosal diseases (including trauma-related injuries); and individuals with other systemic diseases. At the same time, 6 healthy subjects whose gender and age matched those of the HAU patients were selected as the control group.

### Sample Collection

In HAU patients, swabs were collected from specific mucosal areas during both the ulcerative and healing stages: Ulcer phase healthy sites (gingival sulcus: HAU-GS), ulcerated sites (HAU-UC), and healed ulcer sites (HAU-CC). The swabs were placed into a test tube containing 300 μl of DNA extraction buffer, stored at -80°C, and processed in batches. Oral mucosal swab samples from the gingival sulcus were also collected from a normal control group (HC) consisting of healthy individuals. All subjects refrained from eating during the collection of oral swab samples, and venous blood was collected simultaneously to exclude individuals with abnormal blood indicators.

### DNA Extraction and 16S rRNA Gene Amplicon Sequencing

Total genomic DNA was extracted using the CTAB method. PCR amplification targeting the V3-V4 region of the bacterial 16S rRNA genes was performed with the use of specific primers. The forward primer employed was 338F, which has the sequence of 5’-ACTCCTACGGGAGGCAGCA-3’, while the reverse primer used was 806R, with the sequence 5’-GGACTACHVGGGTWTCTAAT-3’. To facilitate multiplex sequencing, unique 7-bp barcodes specific to each sample were added to the primer sequences. The components required for the PCR process included 5 μl of a 5× buffer solution, 0.25 μl of Fast pfu DNA polymerase (with an activity of 5 U/μl), 2 μl of dNTPs at a concentration of 2.5 mM, and 1 μl (at 10 μM concentration) of both the forward and reverse primers. Additionally, 1 μl of the DNA template was included, and the remaining volume was completed with 14.75 μl of double-distilled water.The thermal cycling conditions were set to initiate with an initial denaturation step at 98°C for a duration of 5 min. This was followed by 25 cycles comprising a denaturation phase at 98°C for 30 s, an annealing phase at 53°C for 30 s, and an extension phase at 72°C for 45 s. The cycling concluded with a final extension step at 2°C lasting 5 min to ensure complete amplification. To purify the resulting PCR amplicons, Vazyme VAHTSTM DNA Clean Beads (Vazyme Biotech; Nanjing, China) were utilized, and their concentration was assessed using the Quant-iT PicoGreen dsDNA Assay Kit (Invitrogen; Carlsbad, CA, USA). Following the quantification of each sample, the amplicons were combined in equimolar concentrations. Subsequently, pair-end sequencing was conducted, with a configuration of 2 × 250 bp, utilizing the Illumina NovaSeq platform alongside the NovaSeq 6000 SP Reagent Kit (500 cycles) at the Shanghai Personal Biotechnology (Shanghai, China).

### Species Composition Analysis

The sklearn classifier algorithm in QIIME2 was employed to annotate species using the Greengenes database (http://greengenes.secondgenome.com/). The compositional distribution of each sample at the phylum and genus levels was determined by analyzing the feature table after the removal of singletons. Stacked histograms of the compositional distributions were created using the “ggplot2” package (version 3.5.1) in R language (version 4.3.2).

### Alpha and Beta Diversity Analysis

QIIME2 diversity script was used to perform Alpha and Beta diversity analysis. For Alpha analysis, the minimum flattening depth was set to 10, which encompasses 95% of the samples with the lowest sequencing depth across all samples. Ten depth values were evenly selected between this depth and the minimum depth, with each depth value being flattened 10 times. Subsequently, the Chao1 index, Observed species index, Shannon index, Simpson index, Faith’s PD index, Pielou’s evenness index, and Good’s coverage index were calculated. The “ggplot2” package in R was used to created box plots for Alpha indices, and the Wilcoxon test was used to assess the differences between groups. Differences were deemed statistically significant when p < 0.05.

For Beta diversity analysis, QIIME2 software was used to calculate the Bray-Curtis distance of each sample to form a sample difference distance matrix. The “ape” (version 5.8) function package in R was applied to perform PCoA analysis on the distance matrix.

### Species Diversity Analysis

The “metagenomeSeq” (version 1.43.0) function package in R language was used to compare different sample groups based on linear models, and the species composition differences were analyzed between different groups at the bacterial phylum and genus levels. The “ggplot2” function package in R language was used to draw a Manhattan plot to display the differential bacterial phyla and genera. The Linear discriminant analysis (LDA) effect size combined with LEfSe (LDA Effect Size) analysis of Kruskal-Wallis test and Wilcoxon rank sum test were applied to perform difference analysis on all classification levels of different sample groups simultaneously to identify markers between groups. LDA ≥1 and p<0.05 were used to identify differential microorganisms between different groups.

### Network Analysis

The “igraph” package (version 2.0.3) in R was utilized to construct a correlation matrix for ASV representative sequences with an abundance exceeding 10. Random Matrix Theory (RMT) was employed to establish the filtering threshold for the correlation values. Subsequently, the “ggraph” package in R was used to implement network visualization.

## RESULTS

### Details of Participants

The demographic data of HAU patients and controls included in this study are summarized in Table 1. Three women and three men with HAU were included; the average age was 55.1 ± 11.39 years. The sampling locations of HAU were ulcer center, cure center, and gingival sulcus. The 6 patients in the control group were all men, average age 45.17 ± 13.88 years, and the sampling location was gingival sulcus.

**Table 1:** Demographic data of the control subjects and HAU patients

	Control subjects (N=6)	HAU (N=6)
Age (mean ± SD)	45.17 ± 13.88	55.17 ± 11.39
**Gender**		
Female, n (%)	-	3 (50%)
Male, n (%)	6 (100%)	3 (50%)
**Sampling sites**		
Ulcer center, n (%)	-	6 (100%)
Cure center, n (%)	-	6 (100%)
Gingival sulcus, n (%)	6 (100%)	6 (100%)


### Alpha and Beta Diversities of the Oral Microbiota

**Fig 1:**
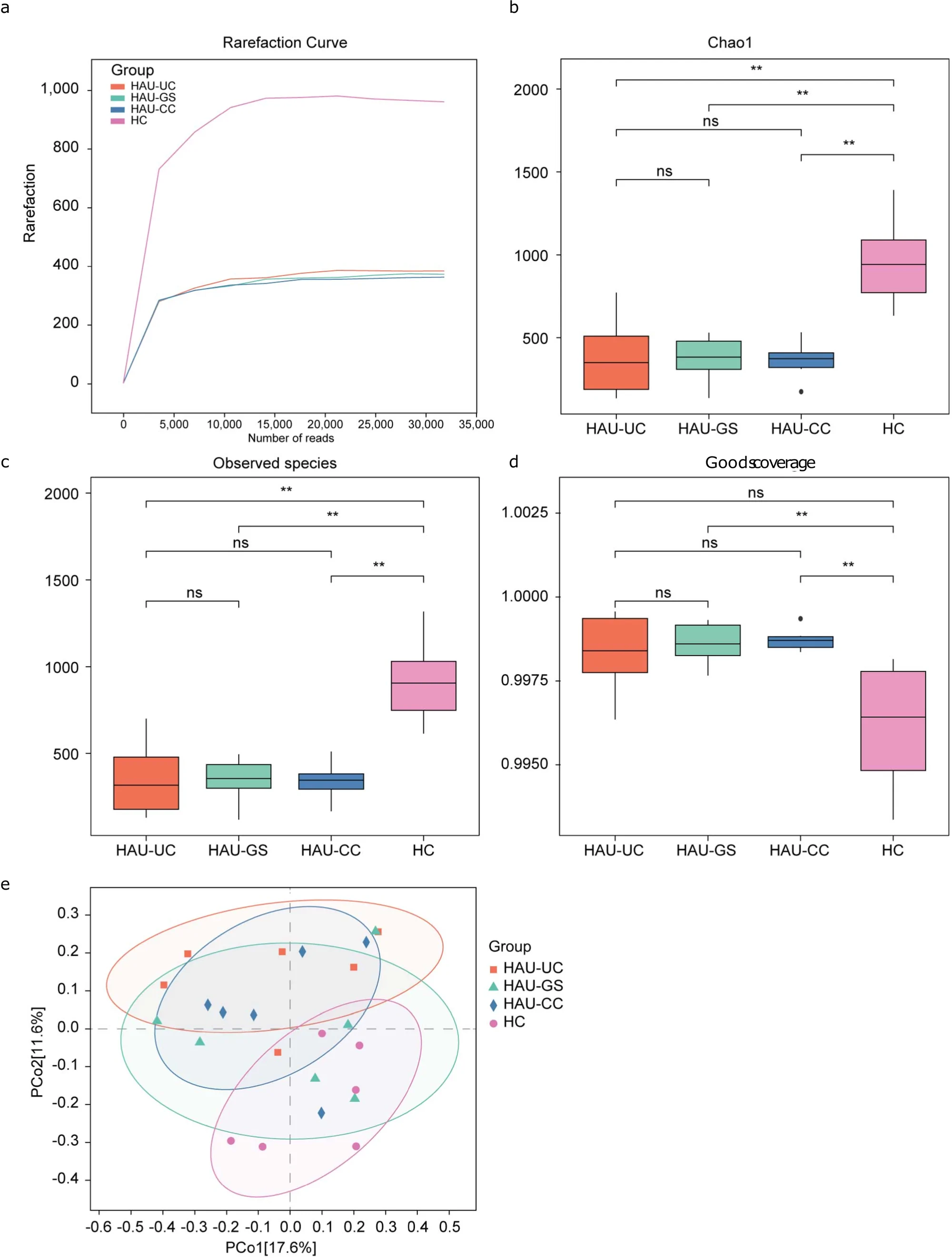
The Alpha and Beta diversities of the oral microbiota. a: sparse curve plot. The horizontal axis represents the number of extracted reads, and the vertical axis represents the number of ASV sequences at this number of reads. b-d: the Chao 1 indexand number of observed species, and Good’s coverage index in four groups. e: results of principal co-ordinates analysis. **p<0.01.

The rarefaction curve was generated using Chao1 to assess species richness. The results indicated that a plateau was reached when the number of reads ranged from 14,154 to 31,846, suggesting that the sequencing depth was sufficient for the research objectives (Fig 1a). Furthermore, we analyzed the alpha diversity among HC, HAU-UC, HAU-GS, and HAU-CC groups. Compared to HC group, the Chao 1 and number of observed species were greatly reduced in HAU-UC, HAU-GS, and HAU-CC groups (Figs 1b and 1c), indicating that the species richness was statistically significantly lower in HAU patients. The diversity and homogeneity among HC, HAU-UC, HAU-GS, and HAU-CC groups were not statistically significantly different (Figs S1A-1D). The HAU-CC and HAU-GS groups had statistically significantly higher coverage than did the HC group (Fig 1d).

Beta diversity analysis of species among groups was conducted using PCoA based on the Bray-Curtis distance matrix. The results indicated that samples from the HAU-UC group and the HC group were separated, while the HAU-GS group was distinct from both the HC group and the HAU-CC group (Fig 1e). Additionally, the diversity within the HAU-UC group was found to be similar (Fig 1e).

### Oral Microbial Species Composition

To characterize the species composition of the HC and the three herpetiform ulcer groups (HAU-UC, HAU-GS, and HAU-CC), we analyzed the top 20 abundances for each group at both the phylum and genus level (Table S1).

**Fig 2:**
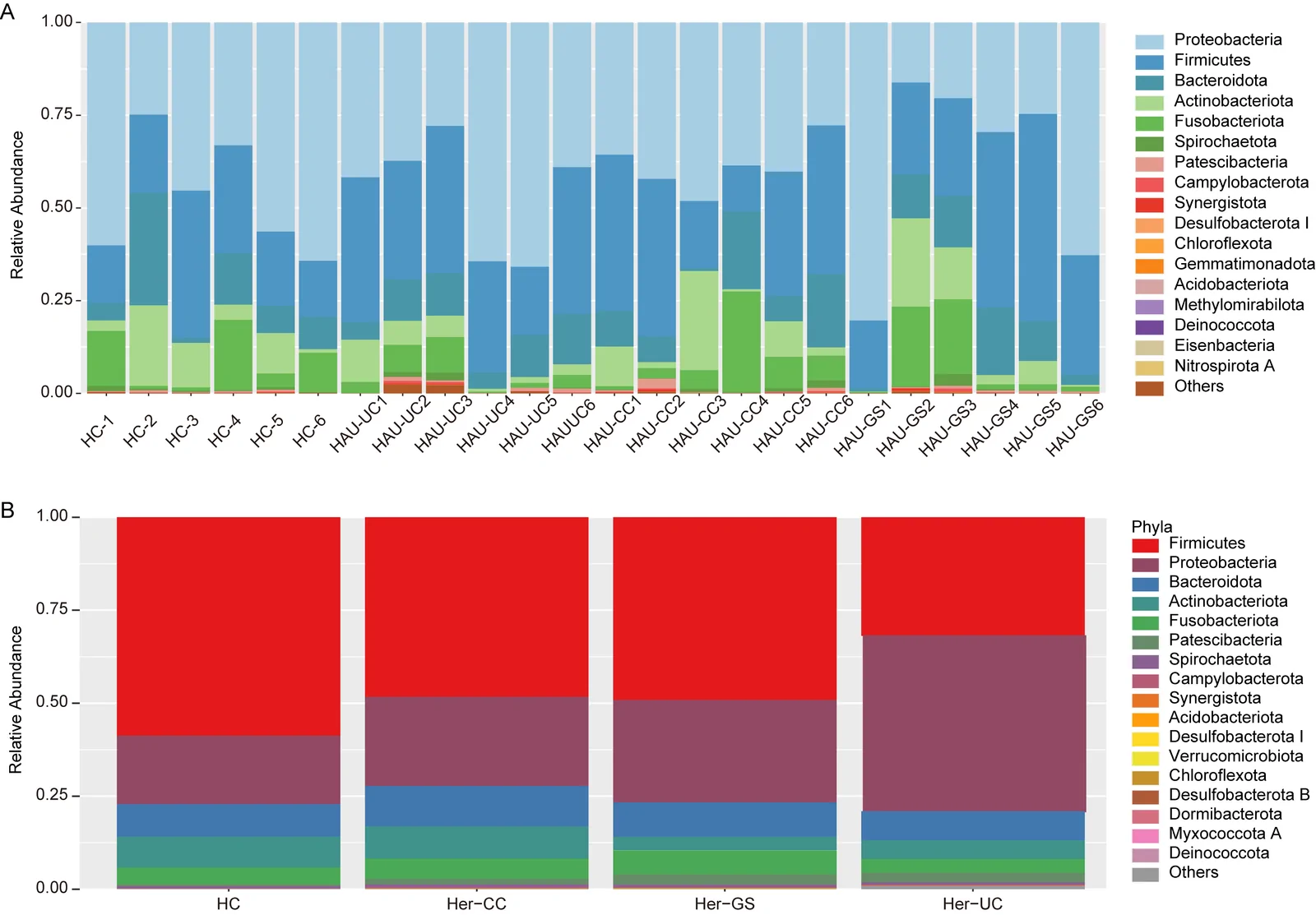
Oral microbial species composition at bacterial phylum level. a: stacked histogram of the abundance of the top 20 bacterial phyla of each sample. b: stacked histogram of the abundance of the bacterial phyla in the four groups.

At the phylum level (Fig 2a), the top four dominant bacterial species of all samples belonge to the phyla Firmicutes, Proteobacteria, Bacteroidota, Actinobacteriota, and Fusobacteriota (Fig 2b). While the composition of the bacterial phylum was similar in the HAU-UC, HAU-GS, HAU-CC, and HC groups, the relative abundances of the same bacteria varied between these groups (Fig 2b). The relative abundances of the top five bacterial phyla in HAU-UC, HAU-GS, HAU-CC, and HC are shown in Table 2. Firmicutes was most abundant in the HC group (0.59 ± 0.11), followed by the HAU-GS (0.50 ± 0.19), HAU-CC (0.49 ± 0.11) and HAU-UC (0.33 ± 0.16) groups. Proteobacteria were less abundant in HC (0.18 ± 0.09), followed by the HAU-CC (0.24 ± 0.13), HAU-GS (0.28 ± 0.14) and HAU-UC groups (0.46 ± 0.14) (Table 2). The abundance of Actinobacteriota in the HC (0.08 ± 0.07) and HAU-CC (0.086 ± 0.09) groups was similar, as well as in the HAU-GS (0.038 ± 0.09) and HAU-UC (0.048 ± 0.04) groups (Table 2).

**Table 2:** The abundance of dominant bacterial phyla in the four groups

Phylum	HC	HAU-GS	HAU-CC	HAU-UC
Firmicutes	0.5872 ± 0.1053	0.4973 ± 0.1931	0.4882 ± 0.1144	0.3308 ± 0.1551
Proteobacteria	0.1842 ± 0.0884	0.2754 ± 0.1426	0.2425 ± 0.1283	0.4605 ± 0.1416
Bacteroidota	0.0873 ± 0.0709	0.0972 ± 0.0612	0.1080 ± 0.0724	0.0948 ± 0.0359
Actinobacteriota	0.0833 ± 0.0702	0.0376 ± 0.0348	0.0859 ± 0.0901	0.048 ± 0.036
Fusobacteriota	0.0447 ± 0.0214	0.0771 ± 0.0933	0.0563 ± 0.0338	0.0415 ± 0.0326


**Fig 3:**
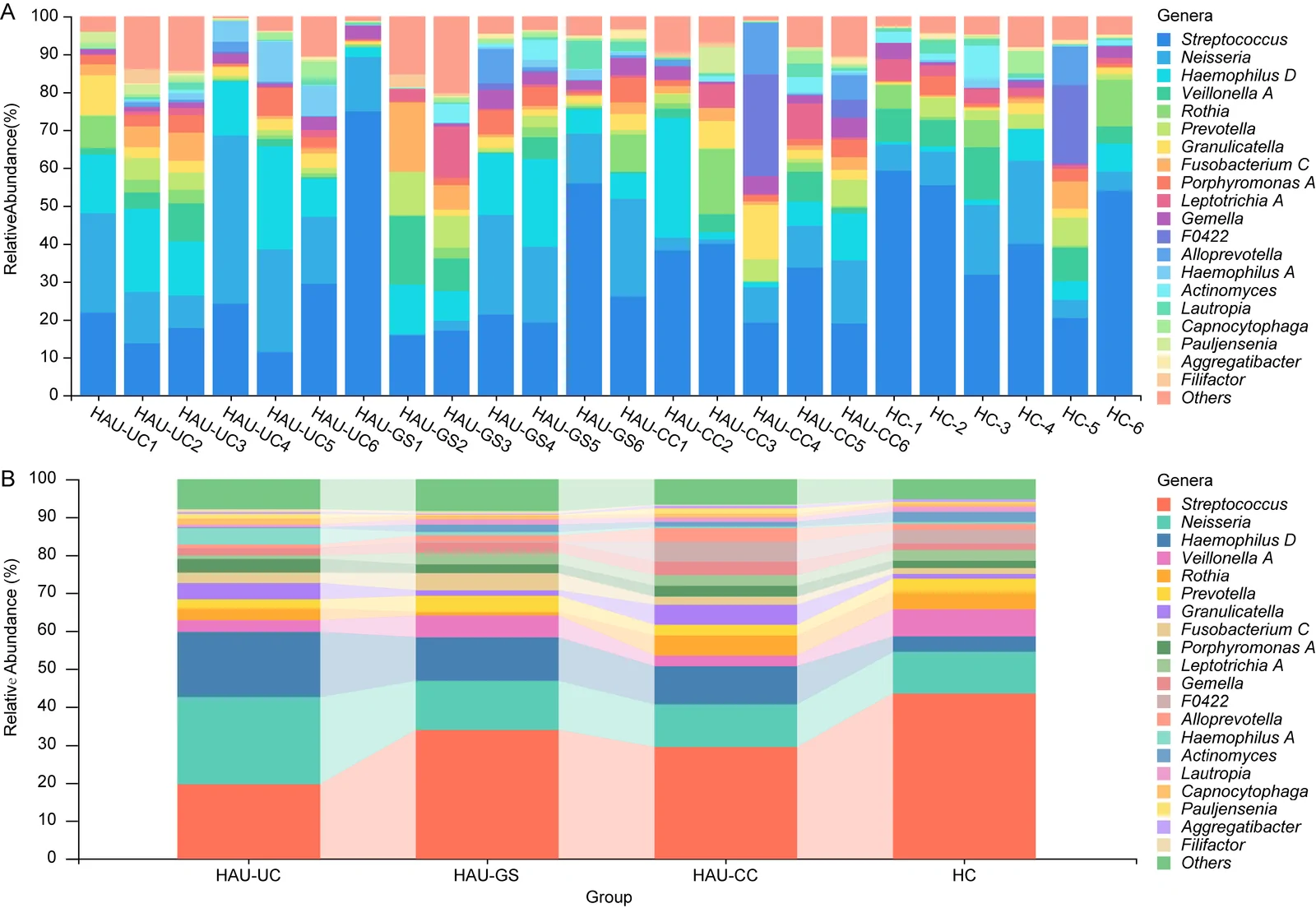
Oral microbial species composition at the bacterial genus level. a: stacked histogram of the abundance of bacterial genera of each sample. b: stacked histogram of the abundance of the bacterial genera in the four groups.

At the genus level (Fig 3a), the dominant bacteria in all samples were *Streptococcus, Neisseria*, and *Haemophilus*_D. The abundance of Streptococcus was highest in the HC group (0.44 ± 0.14), followed by the HAU-CC (0.29 ± 0.08) and the HAU-GS groups (0.34 ± 0.23), while it was lowest in the HAU-UC group (0.20 ± 0.06) (Fig 3b, Table 3). The abundance of *Neisseria * was highest in the HAU-UC group (0.23 ± 0.12). In contrast, the abundance in the HC (0.11 ± 0.07), HAU-CC (0.11 ± 0.08), and HAU-GS groups (0.13 ± 0.09) was found to be similar (Fig 3b, Table 3). The abundance of *Haemophilus*_D was lowest in the HC group (0.04 ± 0.03), comparable in the HAU-CC (0.10 ± 0.10) and HAU-GS groups (0.12 ± 0.07), and highest in the HAU-UC group (0.17 ± 0.06) (Fig 3b, Table 3). Notably, *Pauljensenia *was only observed in the HAU-UC (0.01 ± 0.01) and HAU-CC groups (0.01 ± 0.02).

**Table 3:** The abundance of dominant bacterial genera in the four groups

Taxon	HC	HAU-CC	HAU-GS	HAU-UC
Streptococcus	0.4354 ± 0.1404	0.2942 ± 0.0844	0.3414 ± 0.2292	0.1981 ± 0.0616
Neisseria	0.1094 ± 0.0675	0.1124 ± 0.0819	0.1265 ± 0.0906	0.2288 ± 0.1157
Veillonella_A	0.0716 ± 0.0416	0.0282 ± 0.0265	0.0551 ± 0.0646	0.031 ± 0.0335
Haemophilus_D	0.0405 ± 0.0298	0.1001 ± 0.1029	0.1165 ± 0.0681	0.1717 ± 0.0566
Pauljensenia	0	0.0142 ± 0.0237	0	0.0122 ± 0.0111


### Differences in Oral Microbiota Composition Between the Control and HAU Groups

**Fig 4:**
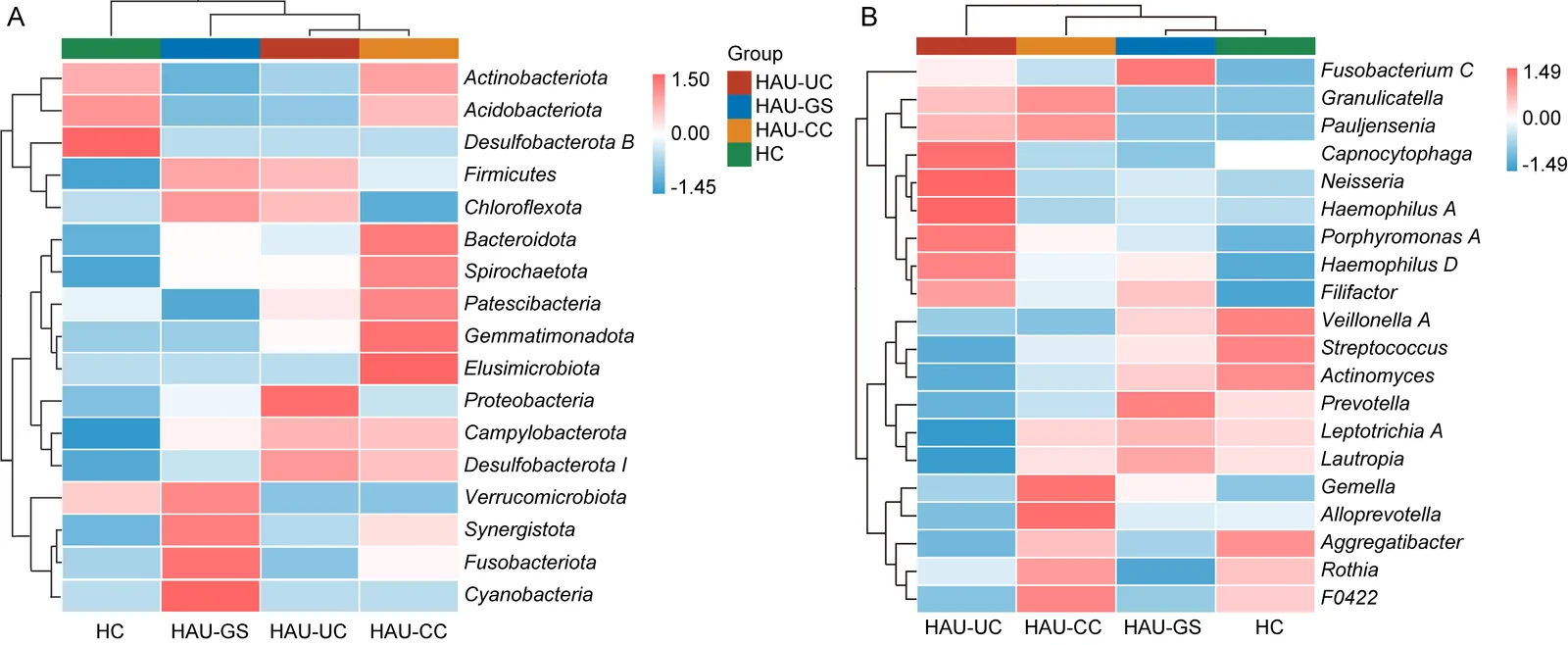
Heat-map of species abundance based on top 20 bacterial phyla (a) and bacterial genera (b) clustering. The color gradients represent species abundance after Z-score normalization. Values <0 indicate abundances below the mean across all samples (with darker blue shades denoting lower relative abundances), while values =0 correspond to the mean abundance (white). Values >0 reflect abundances above the mean (with progressively darker pink hues indicating higher relative abundances). The color scale (dark blue → light blue → white → light pink → dark pink) visually encodes the continuum from low to high relative abundance.

To further compare the differences in species abundance trends among the various sample groups, cluster analysis was conducted using the average abundance of bacterial phyla or genera within each group. At the bacterial phylum level, the species composition of the HAU-UC and HAU-CC groups was similar, whereas the species composition of the HC group was statistically significantly different from both the HAU-UC and HAU-CC groups (Fig 4a). At the bacterial genus level, the species composition of the HC group and the HAU-GS group was similar, whereas the species composition of the HAU-UC group was statistically significantly different from that of the other three groups (Fig 4b).

**Fig 5:**
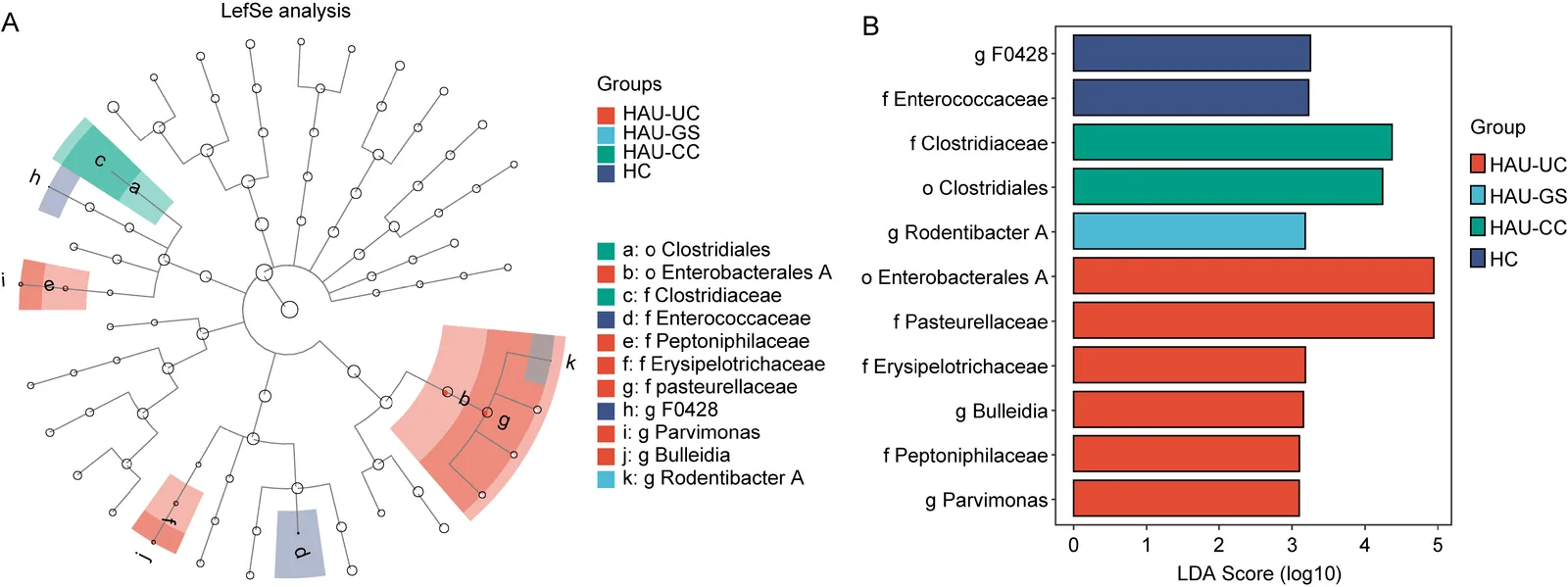
Linear discriminant analysis Effect Size (LEfSe) results. a: LEfSe cladogram; b: LEfSe analysis with linear discriminant analysis.

LEfSe analysis was employed to identify statistically significantly different marker species between the groups. The results indicated that o_*Clostridiales*, o_*Enterobacterales_*A, f_*Clostridiaceae*, f_*Pasteurellaceae*, f_*Erysipelotrichaceae*, f_*Peptoniphilaceae*, and f_*Enterococcaceae*, g_F0428, g_*Parvimonas*, g_*Bulleidia*, and g_*Rodentibacter*_A were identified as marker species among the different groups. Among the different groups. o_*Enterobacterales*_A, f_*Pasteurellaceae, *f_*Erysipelotrichaceae,* g_*Bulleidia*, f_*Peptoniphilaceae,* and g_*Parvimonas *were statistically significantly higher in HAU-UC group compared to normal (HAU-GS and HAU-CC) and HC groups (Figs 5a and 5b). Additionally, g_F0428 and f_*Enterococcaceae *were statistically significantly greater in the oral mucosa of healthy individuals compared to patients with HAU (HAU -UC, HAU-CC, HAU-GS) (Figs 5a and 5b).

### Network Correlation Analysis

**Fig 6:**
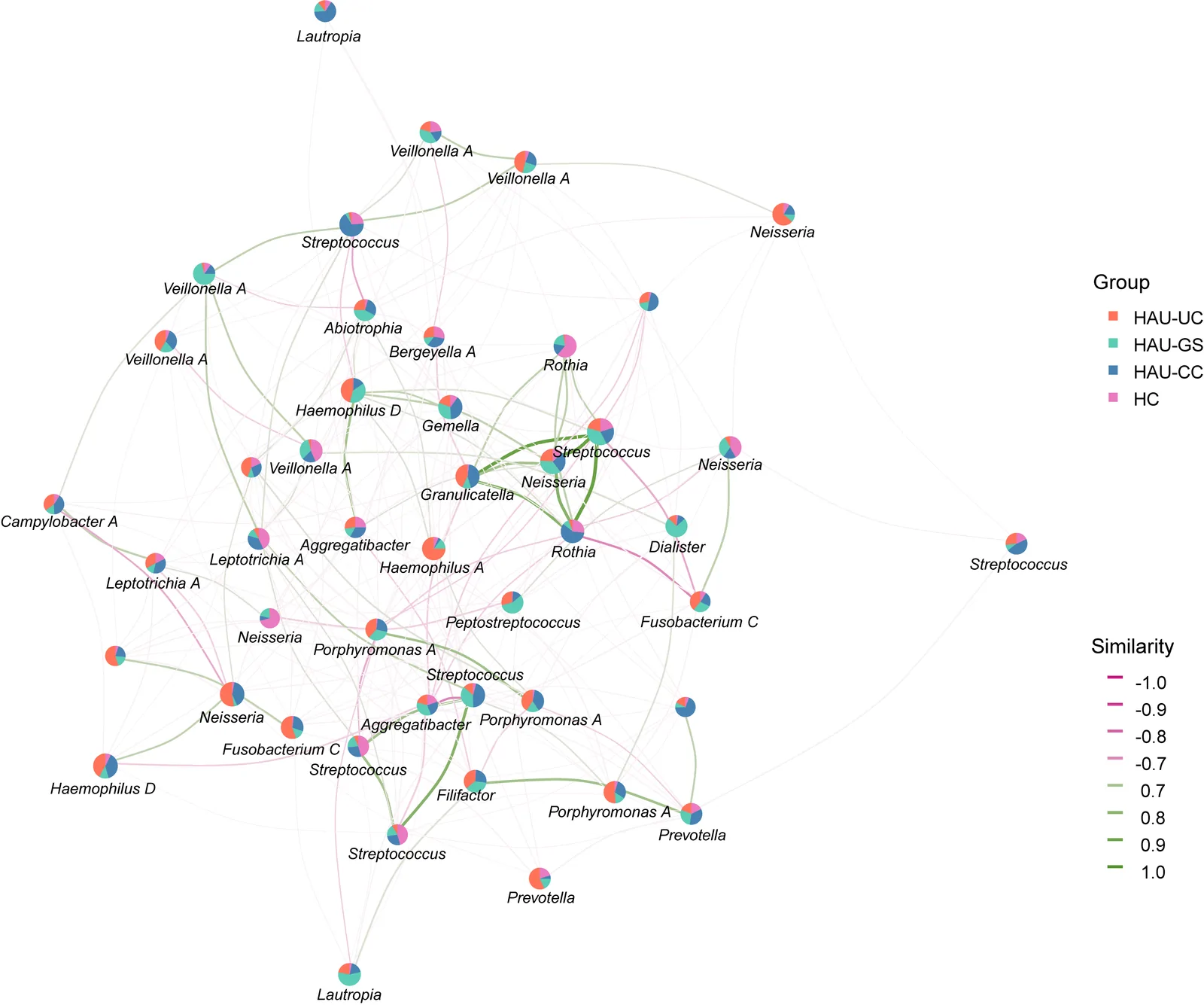
Microbial network analysis results graph. Each node represents a bacterial genus, with the top 20 most abundant genera labeled by their respective names. The color of each node indicates the abundance ratio of the bacterial genus within each group, while the size of the node reflects the total abundance of that genus across the four groups. Edges between nodes represent correlations, with positive correlations depicted in green and negative correlations in pink. The intensity of the color corresponds to the strength of the correlation, with darker shades indicating a greater correlation.

Finally, we screened representative ASV sequences with an abundance greater than 10 and constructed a correlation network diagram. This network comprises 46 nodes and 241 edges, with the bacterial genera containing the representative ASV sequences clearly marked. The three genera with the highest abundance were *Streptococcus, Neisseria*, and *Haemophilus*_D. Furthermore, the ten genera exhibiting the highest betweenness centrality include *Neisseria* (130), *Veillonella*_A (96), *Streptococcus* (86), *Porphyromonas*_A (80), *Gemella *(74), *Aggregatibacter* (59), *Haemophilus*_D (49), *Rothia* (36), *Haemophilus*_A (32), and *Bergeyella*_A (28) (Fig 6).

## DISCUSSION 

Alterations in the mucosal microbiome could contribute to the development of chronic inflammatory mucosal conditions that have traditionally been linked to specific infectious agents.^15^ In the current study, we discovered that imbalances in the mucosal microbiota were correlated with HAU. At the population level, oral flora with higher Alpha diversity exhibited higher resistance to invading microorganisms and antibiotics.^14^ We found that compared to the HC group, the Chao1 index and number of observed species were greatly reduced in HAU-UC, HAU-GS, and HAU-CC groups. This suggested that the oral mucosa of patients with HAU was less resistant to invading pathogens.

The dominant bacterial phyla in the oral mucosa were Firmicutes, Proteobacteria, and Bacteroidota, consistent with previous research results.^24^ The abundance of Firmicutes in the HAU-GS group was lower, while that of Proteobacteria was higher compared to the HC group, suggesting that variations in the relative abundance of these phyla may be associated with the onset and progression of HAU. In the HAU-UC group, the abundance of Firmicutes diminished, and the abundance of Proteobacteria increased relative to the HAU-GS group. Firmicutes are among the predominant phyla in the oral microbial community,^3^ and their reduced abundance may be closely linked to the onset of HAU. Many microorganisms within the Firmicutes exhibit anti-inflammatory and immunomodulatory properties; thus, their decline could result in an immune imbalance within the oral microenvironment.^10^ Furthermore, the increased abundance of Proteobacteria may correlate with the proliferation of certain pathogenic bacteria within this group,^16^ which might considerably contribute to the pathogenesis of HAU. We also found that Firmicutes and Bacteroidetes returned to levels observed in the HAU-GS group in the HAU-CC group. The restoration of Firmicutes abundance may contribute to the stability of the local microecology, while the reduction of Proteobacteria may mitigate inflammatory responses and lower the risk of infection.^20,26^ These results suggested that the changes in the community structure of Firmicutes and Proteobacteria may promote tissue repair in HAU patients.

The dominant bacterial genera in the oral mucosa were *Streptococcus, Neisseria*, and *Haemophilus*_D. *Streptococcus* play a crucial role in oral health by contributing to the maintenance of the oral microbiome’s balance.^5^ However, a decrease in streptococcal populations may create an opportunity for the proliferation of other potentially harmful bacteria. For examples, Kim et al^13^ reported that decreased abundance of *Streptococcus salivarius* and increased of *Acinetobacter johnsonii* was associated with RAS incidence. In ulcer samples, opportunistic pathogens such as *Clostridium* and *Anaerobacteria, Lactobacillus, Cardiobacter*, and *Leptobacter* increased, while *Streptococcus salivarius, Neisseria*, and Bacteroidea decreased.^19^ *Streptococcus* and *Haemophilus* exhibited a statistically significant positive correlation with the skin fungus *Malassezia* in active RAU.^19^ We found that the abundance of *Streptococcus* in the HAU-GS group was lower than that in HC group, whereas *Haemophilus*_D was higher. In the HAU-UC group, the abundance of *Streptococcus* decreased, while the abundances of *Neisseria* and *Haemophilus*_D increased compared to the HAU-GS group. These three genera returned to levels comparable to those in the HAU-GS and HAU-CC groups. The present findings further confirm that the occurrence of herpetic aphthous ulcers was closely linked to an imbalance in oral flora. Specifically, a reduction in the abundance of beneficial bacteria, coupled with an increase in harmful bacteria within the oral mucosa, might contribute to the onset and recurrence of these ulcers.

Furthermore, we found that o_Enterobacterales_A, f_Pasteurellaceae, f_Erysipelotrichaceae, g_Bulleidia, f_Peptoniphilaceae, and g_*Parvimonas* were statistically significantly more abundant in HAU-UC group compared to normal (HAU-GS and HAU-CC) and HC groups. Enterobacteriales have been reported to be more abundant in the intestine of patients with RAU at the taxonomic level of order.^22^ In RAU, the proliferation of harmful bacteria may trigger a local inflammatory response, further damaging the oral mucosa and promoting the emergence of ulcers.^14^ f_Pasteurellaceae includes bacteria that are typically found in the oral cavity and upper respiratory tract.^8^ The Pasteurellaceae family includes a variety of Gram-negative bacteria. Lipopolysaccharides (LPS) from Gram-negative bacteria potently activate Toll-like receptor 4 (TLR4) through the vesicle acidification pathway. At the same time, activation is weakened during the second induction to maintain oral mucosal microbial immune homeostasis.^12,23^ Thus, the increased presence of f_Pasteurellaceae in RAU might be associated with an altered immune response, potentially contributing to the inflammatory processes observed in HAU. *Parvimonas* species have been linked to various inflammatory conditions,^21^ and their increased levels in HAU might be associated with the inflammatory response. They might also contribute to the disruption of the normal oral microbiota, influencing the frequency and severity of HAU episodes. These results suggested that changes in the oral microbiota of patients with HAU might substantially influence both the development and healing of these ulcers. Future research is essential to better elucidate the specific mechanisms underlying these microbial changes and to develop novel strategies for the treatment of HAU.

## CONCLUSION

Our study revealed that imbalances in the mucosal microbiota are correlated with HAU. These findings underscore the distinct microbial signatures associated with HAU and suggest that alterations in microbial communities may may play a role in HAU progression and healing.

### Supplemental material

**Fig S1** Box plot of Alpha diversity index. A, Shannon index. B, Simpson index. C, Faith’s PD index, D, Pielou’s evenness index. ns means no significant difference.:https://www.quintessence-publishing.com/quintessenz/journals/articles/downloads/ohpd_2025_wang_figure_s1.png

**Table S1** The abundance of the top 20 bacterial phyla and genera in the four groups

https://quintessence-publishing.com/quintessenz/journals/articles/downloads/ohpd_2025_wang_table_s1.xls
